# Continuing Professional Development – Radiation Therapy

**DOI:** 10.1002/jmrs.628

**Published:** 2022-11-01

Maximise your CPD by reading the following selected article and answer the five questions. Please remember to self‐claim your CPD and retain your supporting evidence. Answers will be available via the QR code and online at www.asmirt.org/news-and-publications/jmrs, as well as published in JMRS – Volume 70, Issue 4 December 2023.

## Radiation Therapy – Original Article

### What is the effect of a low literacy talking book on patient knowledge, anxiety and communication before radiation therapy starts? A pilot study

Smith‐Lickess SK, Stefanic N, Shaw J, Shepherd H, Naehrig D, Turner RM, Cabrera‐Aguas M, Meiser B, Halkett GKB, Jackson M, Saade G, Bucci J, Milross C, Dhillon HM. (2022) *J Med Radiat Sci*. https://doi.org/10.1002/jmrs.606

1. Participants utilised the talking book according to their preferred learning style. A kinaesthetic learner prefers which of the following?
  - To look at the illustrations
  - To listen to a recording
  - To read the printed text
  - To be ‘hands‐on’ and physically engaged
2. Which of the following was identified as a limitation of this study?
  - The sample size was too small to produce reliable results, more participants are required to increase the statistical power.
  - Many of the study participants did not speak English and could not understand the talking book.
  - This study involved mostly metropolitan‐based English‐speaking women with breast cancer, hence results from this study cannot be generalised across all populations.
  - The participants in this study had such a low health literacy rate that the talking book was difficult to understand.
3. In this study, the talking book provided which of the following?
  - The patients with simplified information with a low‐cost, practical tool.
  - The patients with all the information they needed to know, so that they did not need to ask any questions.
  - The radiation therapists more time to do other duties as they did not need to talk to the patient as much.
  - Improved the education for the nursing staff in the radiation therapy department.
4. Which of the following depicts health literacy?
  - The ability to read medical journals.
  - The ability to search for information on the internet pertaining to health‐related issues.
  - The knowledge, motivation and competence to access and understand and apply health information.
  - The understanding of statistical analysis and research methods.
5. Following on from this study, what are the implementation plans for the talking book?
  - To conduct a larger trial comparing the talking book with standard information.
  - To create an online health literacy training course for radiation oncology healthcare professionals on how to use the talking book in practice.
  - To adapt the talking book for use in Indigenous Aboriginal Australian populations.
  - To make a video for patients to accompany the talking book.

**Recommended further reading:**

1. Halkett G, O'Connor M, Aranda S, Jefford M, Merchant S, York D, Miller L, Schofield P. Communication skills training for radiation therapists: preparing patients for radiation therapy. *J Med Radiat Sci* 2016; 63: 232–41.
2. Smith SK, Zhu Y, Dhillon HM, Milross CG, Taylor J, Halkett G, Zilliacus E. Supporting patients with low health literacy: what role do radiation therapists play? *Support Care Cancer* 2013; 21: 3051–61.
3. Halkett GKB, Merchant S, Smith SK, O'Connor M, Jefford M, Aranda S, Schofield P. Supporting and preparing patients for radiotherapy: patients' and radiation therapists' perspectives on their one‐to‐one consultations. *Eur J Cancer Care (Engl)* 2020; 29: e13284.

## Answers


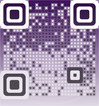


Scan this QR code to find the answers, or visit www.asmirt.org/news-and-publications/jmrs

